# Self-Reported Physical Activity Among Middle-Aged Cancer Survivors in the United States: Behavioral Risk Factor Surveillance System Survey, 2009

**DOI:** 10.5888/pcd11.140067

**Published:** 2014-09-11

**Authors:** Pratibha Nayak, Holly M. Holmes, Hoang T. Nguyen, Linda S. Elting

**Affiliations:** Author Affiliations: Holly M. Holmes, MD, Hoang T. Nguyen, Linda S. Elting, The University of Texas MD Anderson Cancer Center, Houston, Texas.

## Abstract

**Introduction:**

Regular physical activity (PA) can improve health outcomes in cancer survivors, but the rate of adherence to PA recommendations among middle-aged survivors is unclear. We examined adherence to PA recommendations among cancer survivors and controls. We sought to identify correlates of adherence to PA and to determine whether PA adherence is associated with health-related quality of life (HRQOL) among cancer survivors.

**Methods:**

We examined PA adherence among 8,655 cancer survivors and 144,213 control subjects aged 45–64 years who were respondents to the 2009 Behavior Risk Factor Surveillance System survey. We used multinomial logistic regression to assess associations between PA adherence and demographic, psychosocial, and clinical factors, and multivariable linear regression to assess the relationship between PA adherence and HRQOL of cancer survivors.

**Results:**

Cancer survivors and control subjects had similar rates of PA adherence. Of the survivors, 47% met the recommendations of 150 minutes of moderate-intensity PA or 120 minutes of vigorous-intensity PA per week, 41% were somewhat active, and 12% were sedentary. Compared with cancer survivors who were sedentary, survivors who were somewhat active were less likely to be obese (odds ratio [OR], 0.65; *P* < .007), and those who met PA recommendations were less likely to be overweight (OR, 0.61; *P* < .002) or obese (OR, 0.33, *P* < .001). Regression analysis indicated that PA adherence was positively correlated with HRQOL (*P* < .001).

**Conclusion:**

Most cancer survivors did not meet PA recommendations, but those who are active seem to have improved HRQOL. Therefore, targeted interventions to improve adherence to PA among cancer survivors are needed.

## Introduction

The number of cancer survivors living in the United States today is estimated to be more than 13.7 million (1). Among cancer survivors, 35% are middle-aged adults, most commonly with a diagnosis of melanoma or of cancers of the breast, colon, prostate, or cervix. Advances in cancer treatment have improved 5-year survival rates beyond 69% for individuals whose disease was diagnosed at middle age (2); however, these cancer survivors can experience late-onset and long-term effects of treatment, such as radiation injury or cardiovascular disease, 10 or more years after their initial cancer treatment (3). Cancer survivors have increased comorbidity burden, activity limitations, and varied practice of health behaviors (4,5).

Meta-analysis has associated regular physical activity (PA) after cancer diagnosis with reduced adverse effects of cancer treatment (6). Some benefits of PA reported among breast cancer survivors are improved quality of life (7) and decreased anxiety, depression (8), fatigue (9), and risk of developing other chronic diseases (10). Two reports from meta-analyses urged researchers to examine the range and magnitude of positive effects of PA among the diverse population of cancer survivors (7,11).

Most patients with cancer will survive at least 5 years (2), and the positive effects of PA ameliorate the negative consequences of cancer treatment. Therefore, determining the rates of PA adherence and correlates to such adherence is important. This study describes adherence to PA recommendations among cancer survivors compared with control subjects without cancer and the association between survivor adherence to PA guidelines and demographic, psychosocial, and clinical factors, as well as health-related quality of life (HRQOL). Our findings may help public health practitioners design PA interventions for this growing population and ultimately improve quality of life among survivors.

## Methods

We used data from the 2009 Behavioral Risk Factors Surveillance System (BRFSS) survey (12), which is conducted annually among a large, representative sample of noninstitutionalized adults in the United States and US Territories (District of Columbia, Guam, Puerto Rico, and the Virgin Islands). The participants were selected using random-digit–dialing. The core questionnaire consisted of questions on demographics, health behaviors, and chronic conditions.

Respondents were asked about their cancer history as part of the core questionnaire. We used this item to determine cancer survivor and control groups. Cancer survivors were defined as respondents who said yes when asked if they had ever been told by a doctor, nurse, or other health professional that they had cancer or a cancer history. Respondents who reported not having had any type of cancer were considered control subjects. Cancer survivors were asked the number of types of cancers diagnosed, age at diagnosis, and type of cancer.

We restricted the sample for both cancer survivors and control subjects to respondents aged 45–64 years so we could perform an age-matched comparison. We restricted cancer survivors to those who had the most common types of cancer (breast, bladder, cervical, colon, prostate cancer, or melanoma), which allowed for a comparison across PA levels. We excluded cancer survivors who were within 1 year of their cancer diagnoses to eliminate the effect of active treatment on PA.

### Measures

**PA recommendations.** The core questionnaire contains 6 items on PA, including the duration, frequency, and intensity of moderate to vigorous PA per week (Appendix). The survey defines vigorous-intensity PA as activity that causes large increases in breathing or heart rate, and moderate-intensity PA as activity that causes small increases in breathing or heart rate. We used information collected via these survey items to determine adherence to PA guidelines.

We defined adherence to PA guidelines as 150 minutes of moderate-intensity PA or 120 minutes of vigorous-intensity PA per week, as stated by professional medical organizations (13). We categorized PA adherence into 3 groups: meeting guidelines, somewhat active, or sedentary. We defined “meeting guidelines” as engaging in moderate-intensity PA for 30 or more minutes per day on 5 or more days per week, or doing vigorous-intensity PA for 20 or more minutes per day on 3 or more days per week. We categorized as “somewhat active” respondents who reported doing moderate-intensity PA for less than 30 (but more than 10) minutes per day on fewer than 5 days per week or doing vigorous-intensity PA for less than 20 (but more than 10) minutes per day on fewer than 3 days per week. Respondents who reported doing no moderate or vigorous PA were categorized as sedentary.

**Health-related quality of life (HRQOL).** The HRQOL was measured using 3 separate items: poor physical health, poor mental health, and poor overall health. We measured poor physical health with a question about “the number of days during the past 30 days when one’s physical health was not good.” We measured poor mental health with a question about “the number of days during the past 30 days when one’s mental health was not good.” We measured poor overall health with a question about “the number of days during the past 30 days when one’s physical or mental health kept one from doing one’s usual activities.” For each of these items, respondents indicated the total number of days in the previous 30 days when they felt that their physical or mental health was poor or activities were limited. The higher the score, the poorer that person’s HRQOL.

**Demographic, psychosocial, and clinical factors.** Self-reported information was included on sex, race/ethnicity (white, African American, Hispanic, or other), education (more than high school, high school, or less than high school), emotional support (never or always/sometimes), type of cancer (breast, bladder, cervical, colon, prostate, or melanoma), and time since diagnosis (1–2, 2–5, 5–10, or >10 years). Body mass index (BMI) was stratified into 3 categories: normal weight (BMI 18–24.9 kg/m^2^), overweight (BMI 25–29.9 kg/m^2^), or obese (BMI >30 kg/m^2^). We derived the comorbidity count using the single-item questions on comorbid conditions. Respondents were asked whether a health care professional had ever told them they had diabetes, hypertension, arthritis, hyperlipidemia, heart attack, angina, or stroke. We summed the response to heart attack, angina, and stroke into a single binary variable of “cardiovascular diseases.” We coded a response of yes as 1 and a response of no as zero. We summed the scores for the 5 items to create a count of comorbid conditions (0–5). We then categorized the comorbidity count into 3 levels: 0, 1–2, and >2.

To account for the complex survey design of the BRFSS, we used SAS version 9.3 (SAS Institute, Inc) survey procedure with weighted analyses. Cross tabs and chi-squared statistics were used to examine differences in demographic characteristics for categorical variables, and means were used to examine continuous variables. To estimate the adjusted odds ratios and 95% confidence intervals (CIs) for the PA adherence category, we used a multinomial logistic regression, controlling for sex, race or ethnicity, education, emotional support, cancer type, time since diagnosis, BMI, and comorbidity count. We used multivariable linear regression to examine the differences in HRQOL domains (ie, mean difference in number of days of poor physical and mental health) among cancer survivors who met PA guidelines or were somewhat active compared with those who were sedentary, while adjusting for sex, race, education, emotional support, cancer type, time since diagnosis, BMI, and comorbidity count. Significance was set at .05 a priori for all analyses.

## Results

The cancer survivors were older, mostly women, and predominantly white compared with noncancer control subjects (Table 1). The population estimates for comorbidity burden (≥1 comorbidity) were higher among cancer survivors (79%) than among control subjects (69%). No meaningful difference (*P* > .419) in adherence to PA guidelines was noted between cancer survivors and control subjects.

**Table 1:** Cancer Survivors and Noncancer Control Subjects in the United States, Behavioral Risk Factor Surveillance System Survey, 2009

Characteristic	Cancer Survivors		Noncancer Control Subjects	
	Sample Size^a^ = 8,665	Population Estimates (%)	Sample Size^a^ = 144,213	Population Estimates (%)
**Age**				
45–54 years	2,900	1,293,506 (40)	71,365	36,649,702 (58)
**Sex**				
Female	6,285	2,133,893 (66)	85,861	31,394,779 (50)
**Race/ethnicity**				
White	7,407	2,640,178 (82)	116,748	46,379,122 (74)
African American	535	258,049 (8)	11,768	6,077,012 (10)
Hispanic	259	153,028 (5)	7,174	6,275,716 (10)
Other	408	151,802 (5)	7,394	3,575,296 (6)
**Education level**				
High school diploma or less	5,321	1,906,112 (59)	90,197	38,414,000 (61)
**Annual income**				
<25K	1,931	560,953 (19)	28,973	11,528,196 (20)
25–50K	2,038	679,970 (23)	33,394	13,005,105(23)
>50K	3,969	1,707,511 (58)	68,884	32,793,571 (57)
**Body mass index^b^**				
Normal	2,695	1,011,593 (32)	42,461	17,683,155 (29)
Overweight	3,047	1,146,104 (37)	51,651	23,735,150 (39)
Obese	2,626	974,028 (31)	44,542	19,290,183 (32)
**Comorbidity count**				
0	1,758	693,510 (21)	42,261	19,407,186 (31)
1–2	4,858	1,801,207 (56)	76,291	32,961,458 (52)
>2	2,049	733,592 (23)	25,659	10,448,666 (17)
**Physical activity levels^c^**				
Meet physical activity guidelines	4,068	1,518,975 (47)	68,570	30,003,912 (48)
Somewhat active	3,448	1,329,279 (41)	57,368	25,123,116 (40)
Sedentary	1,149	380,054 (12)	18,275	7,691,285 (12)

a Sample size refers to the observed number of units in the sample that share a given characteristic, and population estimate is the corresponding estimated number in the population.

b Body mass index, calculated as weight (kg)/[height (m)]^2^_,_ was stratified into 3 categories: normal weight (BMI 18.0–24.9 kg/m^2^), overweight (BMI 25.0–29.9 kg/m^2^), or obese (BMI >30.0 kg/m^2^).

c Physical activity was categorized as meeting guidelines (>120–150 min/wk), somewhat active (11–149 min/wk) and sedentary (no physical activity).

On the basis of population estimates, nearly 60% of cancer survivors were aged 55 to 64 years, and most (66%) were female, white (82%), and had a high school diploma or less education (59%). Breast cancer survivors made up 35% of the sample population, and more than 10 years had passed since the cancer diagnosis for nearly 40% of the cancer survivors. Most (79%) self-reported having at least 1 comorbid condition, and 68% reported being overweight or obese. Adherence to PA guidelines was suboptimal among middle-aged cancer survivors in the United States; only 47% met PA recommendations (Table 2).

**Table 2:** US Cancer Survivors by Physical Activity Levels^a^, Behavioral Risk Factor Surveillance System Survey, 2009

Characteristic	Met Physical Activity Guidelines		Somewhat Active		Sedentary		*P* Value
	Sample Size^b^, 4,068	Population Estimate^b^, N = 1,518,975 (%)	Sample Size^b^, 3,448	Population Estimate^b^, N = 1,329,279 (%)	Sample Size^b^, 1,149	Population Estimate^b^, N = 380,054 (%)	
**Age**							
45–54 years	1,416	615,646 (41)	1,128	544,232 (41)	356	133,629 (35)	.142
**Sex**							
Female	2,867	977,764 (64)	2,536	877,579 (66)	882	278,551 (73)	.01
**Race/ethnicity**							
White	3,577	1,287,844 (85)	2,918	1,058,431 (80)	912	293,902 (78)	<.001
African American	162	80,571(5)	260	133,839 (10)	113	43,638 (12)	
Hispanic	115	83,689 (6)	106	57,625 (4)	38	11,715 (3)	
Other	182	55,511 (4)	149	70,566 (5)	77	25,725 (7)	
**Education **							
High school diploma or less	2,221	800,699 (53)	2,242	830,823 (63)	858	274,589 (72)	<.001
**Emotional support**							
Has emotional support	3,380	1,275,397(84)	2,637	1,031,578 (78)	763	255,125 (68)	<.001
**Cancer type**							
Breast	1,521	525,112 (35)	1,306	468,369 (35)	420	134,032 (35)	<.001
Bladder	109	34,712 (2)	87	28,128 (2)	40	17,788 (5)	
Cervical	651	232,892 (15)	640	215,778 (16)	277	92,041 (24)	
Colon	288	111,940 (7)	297	121,518 (9)	124	38,651 (10)	
Melanoma	1,022	402,084 (27)	718	285,606 (22)	189	63,666 (17)	
Prostate	477	212,234 (14)	400	209,881 (16)	99	33,876 (9)	
**Time since diagnosis**							
1–2 years	715	255,399 (17)	604	266,235 (20)	206	75,028 (20)	.006
2–5 years	778	328,675 (22)	674	264,249 (20)	190	50,179 (13)	
5–10 years	902	344,384 (23)	745	302,485 (23)	232	83,842 (22)	
>10 years	1,673	590,517 (39)	1,425	496,311 (37)	521	171,005 (45)	
**Body mass index^c^**							
Normal	1,520	575,429 (39)	925	364,649 (28)	250	71,514 (20)	<.001
Overweight	1,493	573,138 (39)	1,221	452,601 (35)	333	120,365 (34)	
Obese	943	335,607 (23)	1,168	471,011 (37)	515	167,410 (47)	
**Comorbidity count**							
0	968	371,236 (24)	650	275,474 (21)	140	46,800 (12)	<.001
1–2	2,389	892,917 (59)	1,916	719,210 (54)	553	189,080 (50)	
>2	711	254,822 (17)	882	334,595 (25)	456	144,175 (38)	

a Physical activity was categorized as meeting guidelines (>120–150 min/wk), somewhat active (11–149 min/wk), and sedentary (no physical activity).

b Sample size refers to the observed number of units in the sample that share a given characteristic, and population estimate is the corresponding estimated number in the population.

c Body mass index was categorized as normal weight (BMI 18.0–24.9 kg/m^2^), overweight (BMI 25.0–29.9 kg/m^2^), or obese (BMI >30.0 kg/m^2^).

After adjusting for demographic and medical factors, we found many covariates to be independently associated with a sedentary lifestyle in the multinomial regression model (Table 3). Male survivors were more likely to meet PA recommendations than female survivors. Sedentary activity was significantly associated with race; African Americans and “other” racial categories were less likely to meet PA recommendations than whites. Having a college degree was significantly associated with meeting PA guidelines. A lack of emotional support, being obese, and having more than 2 comorbidities all significantly (*P* values <.05) increased the odds of being sedentary (Table 3). Cancer survivors whose cancer had been diagnosed 2 to 5 years previously were twice as likely to meet PA recommendations as survivors whose cancer was diagnosed 1 to 2 years previously.

**Table 3:** Multinomial Logistic Regression Model of Adherence to Physical Activity and Selected Factors Among US Cancer Survivors, Behavioral Risk Factor Surveillance System Survey, 2009

Characteristic	Sedentary^a^ vs Met Physical Activity Guidelines^a^			Sedentary^a^ vs Insufficient Activity^a^		
	AOR (95% CI)	*P* Value		AOR (95% CI)		*P* Value
**Sex**						
Female	1 [Reference]	—	1 [Reference]			—
Male	1.52 (1.01–2.29)	.043	1.13 (0.76–1.68)			.545
**Race/Ethnicity**						
White	1 [Reference]	—		1 [Reference]		—
African American	0.58 (0.34–0.99)	.047		0.93 (0.57–1.52)		.769
Hispanic	2.05 (1.04–4.02)	.038		1.44 (0.73–2.85)		.292
Other	0.53 (0.31–0.93)	.026		0.80 (0.45–1.42)		.445
**Education**						
High school diploma or less	1 [Reference]	—		1 [Reference]		—
College graduate	1.56 (1.19–2.04)	.001		1.18 (0.90–1.55)		.232
**Emotional support**						
Never	1 [Reference]	—		1 [Reference]		—
Always	2.00 (1.51–2.62)	<.001		1.41 (1.08–1.83)		.011
**Cancer type**						
Breast	1 [Reference]	—		1 [Reference]		—
Bladder	0.46 (0.24–0.89)	.022		0.51 (0.25–1.01)		.052
Cervical	0.83 (0.58–1.19)	.317		0.81 (0.57–1.15)		.243
Colon	0.74 (0.46–1.19)	.216		0.97 (0.62–1.51)		.891
Melanoma	1.34 (0.91–1.97)	.137		1.26 (0.86–1.85)		.240
Prostate	1.23 (0.67–2.24)	.506		1.70 (0.93–3.09)		.084
**Time since diagnosis, y**						
1–2	1 [Reference]	—		1 [Reference]		—
2–5	1.95 (1.31–2.91)	.001		1.53 (1.02–2.29)		.039
5–10	1.25 (0.86–1.84)	.248		1.13 (0.76–1.67)		.550
>10	1.31 (0.90–1.92)	.157		1.11 (0.75–1.63)		.607
**BMI^b^**						
Normal	1 [Reference]	—		1 [Reference]		—
Overweight	0.61 (0.45–0.84)	.002		0.75 (0.54–1.04)		.086
Obese	0.33 (0.24–0.44)	<.001		0.65 (0.48–0.89)		.007
**Comorbidity count**						
0	1 [Reference]	—			1 [Reference]	—
1–2	0.74 (0.53–1.03)	.072			.69 (0.49–0.97)	.035
>2	0.38 (0.26–0.55)	<.001			0.48 (0.33–0.69)	<.001

Abbreviations: AOR, adjusted odds ratio; CI, confidence interval; —, no *P* values for Reference category.

a Physical activity was categorized as meeting guidelines (>120–150 min/wk), somewhat active (11–149 min/wk), and sedentary (no physical activity).

b Body mass index (weight (kg)/[height (m)]^2^) was categorized as normal weight (BMI 18.0–24.9 kg/m^2^), overweight (BMI 25.0–29.9 kg/m^2^), or obese (BMI >30.0 kg/m^2^).

HRQOL was associated with adherence to PA recommendations. The mean number of days with poor physical health was 3.95 days (standard error [SE], 0.22) for cancer survivors who met PA guidelines, 5.48 days (SE, 0.29) for somewhat active survivors, and 13.51 days (SE, 0.64) for sedentary survivors (Figure). We observed a similar trend for the number of days with poor mental health: 3.55 days (SE, 0.25) for cancer survivors who met PA guidelines, 4.29 days (SE, 0.24) for those who were somewhat active, and 7.96 days (SE, 0.55) for sedentary cancer survivors. We observed a dose–response pattern across levels of PA adherence and HRQOL. Results from the multivariable linear regression indicated that increasing levels of PA adherence were significantly associated with improved HRQOL when controlling for demographic, psychosocial, and medical factors (*P* < .001).

**Figure:**
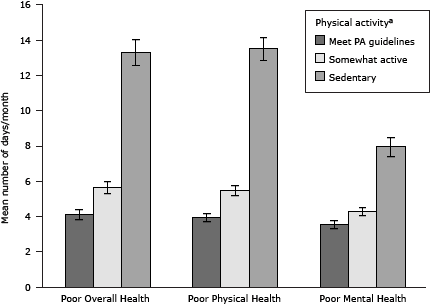
Association between health-related quality of life and physical activity levels among cancer survivors in the United States, Behavioral Risk Factor Surveillance System Survey, 2009. The higher the score, the poorer a person’s health-related quality of life. The error bar represents the 95% confidence interval of the mean number of days per month. Among cancer survivors, the higher the level of physical activity adherence, the fewer days with poor health-related quality of life across all 3 items (poor overall health, poor physical health, or poor mental health). **Table:** 

Level of Physical Activity	Poor Overall Health	Poor Physical Health	Poor Mental Health
Sedentary	13.3 (11.81–14.8)	13.51 (12.2–14.77)	7.96 (6.88–9.05)
Somewhat active	5.64 (4.97–6.31)	5.48 (4.91–6.04)	4.29 (3.82–4.76)
Meets guidelines	4.12 (3.56–4.69)	3.95 (3.52–4.39)	3.55 (3.07–4.04)

## Discussion

Using data from the nationally representative BRFSS 2009 survey, we found suboptimal PA adherence among middle-aged cancer survivors, which was similar to a related finding for the general population (5,14); however, because of their increased risk of comorbidity (8) and cancer recurrence (15), it is more important for cancer survivors to adhere to PA recommendations.

Some population-based studies indicated that cancer survivors perform less PA compared to the general population (16,17). Our findings concur with those of 2 other studies that compared cancer survivors and controls (18,19). These differences may result from variations in populations, study design, or the ways in which PA is assessed. Coups et al and Bellizi et al respectively reported that 25% and 33% of cancer survivors aged 40–64 years met PA guidelines (5,16). Richardson et al found that 46% of such cancer survivors reported leisure PA similar to our study results (17). Previous studies included people diagnosed with cancer, irrespective of cancer type, whereas we restricted our sample to respondents diagnosed with melanoma, or breast, prostate, cervical, bladder, or colon cancer (18,19). Kwon et al indicated that PA among cancer survivors varied across cancer type (14), which may be due to treatment variation, predisposition to obesity, and other comorbid conditions. 

### Correlates for adherence to physical activity guidelines

Approximately half of the cancer survivors aged 45–64 years in the BRFSS 2009 survey met PA guidelines, and 41% reported a lower level of PA. Our study describes the characteristics of this large group of survivors with inadequate PA who may benefit from targeted interventions.

Cancer survivors who are sedentary were more likely to be female, African American, have lower education levels, or have low levels of emotional support (19,20). Having others who motivate the survivor or offer support in dealing with ongoing psychological distress lessens barriers to participation in PA (20). A weight loss intervention called “Moving Forward,” which was designed for African American breast cancer survivors, found that positive social support from friends and family encouraged survivors to maintain high levels of participation and retention in the weight loss program (21). The intervention included the participation of friends and family and promoted the interdependence of culture and kinship networks to support personal health decisions. The success of this intervention demonstrates the role of social support in promoting PA participation and how tailored programs can encourage cancer survivors who are overweight or obese to adhere to PA guidelines.

Our study found that cancer survivors who were sedentary had an increased burden of comorbidity and obesity. These findings concur with those of previous studies: being overweight or obese was significantly associated with not meeting PA guidelines (22,23). Along with targeted interventions to increase PA among sedentary cancer survivors, clinical management of obesity or other comorbidities may be necessary before initiating such programs.

### Health-related quality of life and physical activity

High levels of HRQOL was associated with high levels of adherence to PA guidelines, and cancer survivors who were somewhat active reported better HRQOL than did sedentary cancer survivors, which agreed with previous findings (24). Cancer survivors who practiced positive lifestyle behaviors reported high HRQOL, particularly those who met the PA guidelines (24).

Sedentary survivors reported experiencing more recent days of poor health compared with cancer survivors who reported being somewhat active or who met PA guidelines. Previous studies examined this relationship only among individuals with colorectal cancer and non-Hodgkin’s lymphoma (25,26). PA promotion should motivate sedentary cancer survivors to become more active so they can improve their HRQOL. Many cancer survivors may be more willing to initiate a less intense exercise routine than to immediately try to meet PA guidelines, and our study findings indicate that small doses of PA are associated with improved HRQOL. Cancer survivors should be encouraged to incorporate some form of PA into their daily life.

This study has strengths and limitations. First, the 2009 BRFSS collected information using landline telephone interviews of noninstitutionalized individuals; therefore, it did not represent people who are without landline telephones, use cellular telephones exclusively, or are in institutions. However, the BRFSS survey is representative of noninstitutionalized adults living in the United States (covers all US states and territories) and thus enables researchers to estimate the national prevalence of health-related behaviors such as PA. Second, the information collected is self-reported by the respondents and thus is subject to recall bias or inaccuracy. Furthermore, BRFSS does not collect information on the type of medical treatments received or their duration. To address bias arising from limitations in participation caused by aggressive treatment effects, we restricted the study sample to participants who were at least 1 year beyond their cancer diagnosis. Third, we did not include income in the adjusted model because of the large number of nonresponses. Socioeconomic status plays an important role in education level, access to recreational facilities, and availability of leisure time to engage in PA. To reduce this bias, we included surrogate variables of socioeconomic status such as education and race. Finally, because BRFSS is a cross-sectional survey, we can assess only association and not causality.

Our study has several strengths. We examined adherence to PA among cancer survivors of middle age (an understudied group) across diverse cancer types and using a population-based sample. Most previous studies examined adherence to PA as meeting versus not meeting guidelines. Such broad categories do not allow for an examination of the influences faced by cancer survivors who are somewhat active but do not meet the guidelines. We examined PA adherence on 3 levels: meeting guidelines, somewhat active, and sedentary. This approach allowed us to examine the relationship between varying levels of PA adherence and HRQOL. Finally, most previous studies focused on short-term rather than long-term cancer survivors. We studied cancer survivors who received their diagnosis at least 1 year before the study, and 40% of the survivors were more than 10 years post diagnosis.

This study highlights the subgroup of cancer survivors who are active but are not meeting PA guidelines and who may benefit from an intervention that helps them incorporate more PA into their daily life. Cancer survivors who are obese and have more than 2 comorbidities are likely to be sedentary. This subgroup of cancer survivors needs to be targeted and encouraged to incorporate some PA into their daily life. Previous research indicated that primary care providers have a positive influence on a patient’s PA behavior and that provider-based counseling improves adherence to PA guidelines among sedentary individuals (27). Despite this, few physicians recommend PA to the cancer survivors among their patients (28). Some barriers reported by physicians were concerns about safety and a lack of time during office visits. The American College of Sports Medicine’s roundtable on exercise guidelines for cancer survivors indicated that PA is safe for this population and should be undertaken even during active disease and treatment (29). Primary care physicians are in the best position to address obesity and coexisting medical conditions that may limit their patients’ ability to meet recommended PA guidelines. Because HRQOL improves dramatically with PA, even levels not sufficient to meet the guidelines can be helpful for cancer survivors. A one-size-fits-all approach may not work with this group, and based on their current activity levels and health status, such patients may need a PA intervention program that helps them meet their unique needs.

## Appendix: Behavioral Risk Factor Surveillance System Survey, 2009 — Questions on Physical Activity

This file is available for download as a Microsoft Word document.
