# Supplementary material for: Self-Reported Physical Activity Among Middle-Aged Cancer Survivors in the United States: Behavioral Risk Factor Surveillance System Survey, 2009
Source: Prev Chronic Dis. 2014 Sep 11;11:E156. doi: 10.5888/pcd11.140067 (PMC4165550; doi:10.5888/pcd11.140067)
Supplement: Supplementary file 1 [file 14_0067_01.docx]

**Appendix.** Behavioral Risk Factor Surveillance System Survey, 2009 — questions on physical activity

| Question | Response option |
| --- | --- |
| **Moderate activity** |  |
| 1. Now, thinking about the moderate activities you do in a usual week, do you do moderate activities for at least 10 minutes at a time, such as brisk walking, bicycling, vacuuming, gardening, or anything else that causes some increase in breathing or heart rate? | Yes/No/ Don’t know/Refused |
| 1. How many days per week do you do these moderate activities for at least 10 minutes at a time? | _ _ Days per week |
| 1. On days when you do moderate activities for at least 10 minutes at a time, how much total time per day do you spend doing these activities? | _:_ _ Hours and minutes per day |
| **Vigorous Activity** |  |
| 1. Now, thinking about the vigorous activities you do in a usual week, do you do vigorous activities for at least 10 minutes at a time, such as running, aerobics, heavy yard work, or anything else that causes large increases in breathing or heart rate? | Yes/No/ Don’t know/Refused |
| 1. How many days per week do you do these vigorous activities for at least 10 minutes at a time? | _ _ Days per week |
| 1. On days when you do vigorous activities for at least 10 minutes at a time, how much total time per day do you spend doing these activities? | _:_ _ Hours and minutes per day |

Source: Centers for Disease Control and Prevention (CDC). Behavioral Risk Factor Surveillance System Survey Questionnaire. Atlanta (GA): U.S. Department of Health and Human Services, Centers for Disease Control and Prevention; 2009.
